# Stimulus-Specific Adaptation in the Auditory Thalamus of the Anesthetized Rat

**DOI:** 10.1371/journal.pone.0014071

**Published:** 2010-11-19

**Authors:** Flora M. Antunes, Israel Nelken, Ellen Covey, Manuel S. Malmierca

**Affiliations:** 1 Auditory Neurophysiology Unit, Laboratory for the Neurobiology of Hearing, Institute of Neuroscience of Castilla y León, University of Salamanca, Salamanca, Spain; 2 Department of Cell Biology and Pathology, Faculty of Medicine, University of Salamanca, Salamanca, Spain; 3 Department of Neurobiology, Institute of Life Sciences, The Hebrew University of Jerusalem, Jerusalem, Israel; 4 The Edmond and Lily Safra Center for Brain Sciences, The Hebrew University of Jerusalem, Jerusalem, Israel; 5 Department of Psychology, University of Washington, Seattle, Washington, United States of America; Hotchkiss Brain Institute, University of Calgary, Canada

## Abstract

The specific adaptation of neuronal responses to a repeated stimulus (Stimulus-specific adaptation, SSA), which does not fully generalize to other stimuli, provides a mechanism for emphasizing rare and potentially interesting sensory events. Previous studies have demonstrated that neurons in the auditory cortex and inferior colliculus show SSA. However, the contribution of the medial geniculate body (MGB) and its main subdivisions to SSA and detection of rare sounds remains poorly characterized. We recorded from single neurons in the MGB of anaesthetized rats while presenting a sequence composed of a rare tone presented in the context of a common tone (oddball sequences). We demonstrate that a significant percentage of neurons in MGB adapt in a stimulus-specific manner. Neurons in the medial and dorsal subdivisions showed the strongest SSA, linking this property to the non-lemniscal pathway. Some neurons in the non-lemniscal regions showed strong SSA even under extreme testing conditions (*e.g*., a frequency interval of 0.14 octaves combined with a stimulus onset asynchrony of 2000 ms). Some of these neurons were able to discriminate between two very close frequencies (frequency interval of 0.057 octaves), revealing evidence of hyperacuity in neurons at a subcortical level. Thus, SSA is expressed strongly in the rat auditory thalamus and contribute significantly to auditory change detection.

## Introduction

Rare sounds may indicate events of behavioural importance to which an individual should attend for survival. On the other hand, repeating sounds without behavioural consequences can be assumed to be unimportant. Indeed, neurons at several levels of the auditory system have been shown to signal the occurrence of rare sounds while reducing their responses to repeated ones. The specific adaptation to repeated sounds, which does not generalize to other sounds, is referred to as stimulus-specific adaptation (SSA). Recent SSA studies [1]–[13] have revealed that SSA in single auditory neurons shares many similarities with Mismatch Negativity (MMN) [5], [14]–[18], and may contribute to auditory scene analysis [19].

The medial geniculate body (MGB) is the principal nucleus of the auditory thalamus and possesses three main subdivisions: ventral (MGV), dorsal (MGD) and medial (MGM) [20]–[22]. The non-lemniscal divisions are morphologically and functionally different from the lemniscal MGV [21], [23]–[25]. The MGV constitutes the lemniscal part of the auditory thalamus and is thought to process basic acoustic features of the stimulus, whereas the MGD and MGM comprise the non-lemniscal part, and are thought to process more complex features.

The MGB receives ascending inputs from the inferior colliculus (IC) [21], [26], [27] and massive descending inputs from the auditory cortex (AC; [21], [28]–[32]. SSA is known to be present in the thalamus: Yu and colleagues [13] demonstrated strong SSA in the reticular nucleus. However, a previous report of SSA in the MGB of mice [1] showed substantially weaker levels of adaptation than those found in AC [10]–[12] or IC [4] neurons. Given its connections with the AC and the IC, one would expect neurons in the MGB to show strong SSA as well. In particular, the differences in the SSA exhibited by neurons in the three main subdivisions of the MGB need to be clarified. The mouse MGB study [1] showed SSA in the MGM and in the lemniscal MGV but not in the non-lemniscal MGD subdivision. Nevertheless, the studies of the rat IC [4], [6] demonstrated stronger SSA in the non-lemniscal regions than in the lemniscal central nucleus.

Here, using the same oddball paradigm as previously used in AC and IC studies, we recorded from single neurons throughout the rat MGB. We aimed to characterize SSA in its main subdivisions under several conditions hitherto unexplored. Our results demonstrate that MGB neurons exhibit SSA levels as high as those found in the IC and AC. Furthermore, SSA is more prominent in the medial and dorsal subdivisions, linking this property to the non-lemniscal auditory pathway. Thus, we demonstrate that the MGB has substantial SSA and strongly represents frequency change detection. Preliminary results have been presented elsewhere [33]–[35].

## Results

To look for evidence of SSA in the MGB we recorded the responses of single neurons (n = 93) while presenting sequences of tones with two different frequencies (f_1_ and f_2_; 400 stimulus presentations) in which the standard and deviant stimuli occurred at different probability ratios (90/10% or 70/30%), with different frequency intervals (Δf = 0.37, 0.10 or 0.04) and at different repetition rates (SOA = 2000 ms, 500 ms, 250 ms or 125 ms). We localized 60 of the 93 units recorded to one of the three main MGB subdivisions: 24 units were localized to the MGD; 18 to the MGM; and 18 to the MGV. The remaining units could not be localized with confidence and included units close to the borders between subdivisions (15 out of 33).

**Figure 1:**
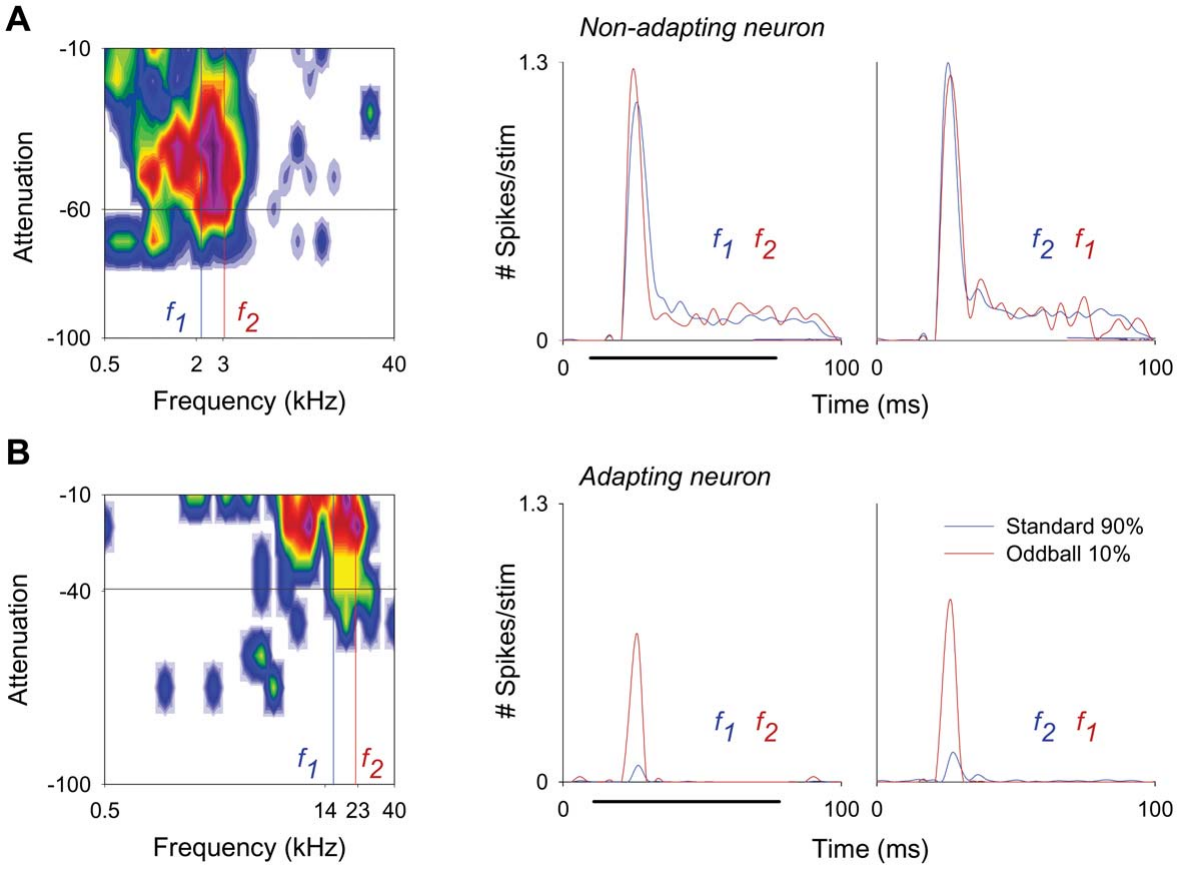
Some MGB neurons exhibit extreme levels of stimulus-specific adaptation. Response of two neurons to pure-tone stimuli of two frequencies (f1 and f2), selected from within the frequency response area (left panels), presented in an oddball paradigm (SOA = 500 ms; *Δf* = 0.37). Red and blue lines in the peri-stimulus time histograms (PSTHs; second and third panels) represent the neuronal activity (number of spikes/stimulus; bin duration: 3 ms; number of bins: 168) elicited by the deviant tone (10% probability) and standard tone (90% probability) respectively, in the first block of stimuli (left PSTHs; f1 as standard, f2 as deviant) and in the second block of stimuli (right PSTHs; f2 as standard, f1 as deviant). Black horizontal lines below the PSTHs in the second panels indicate the duration of the stimulus (75 ms). The non-adapting neuron (A) has a similar response to the standard and to the deviant frequencies in both blocks of stimuli. In contrast, the adapting neuron (B) shows a much stronger response to the deviant than to the standard frequency in both blocks of stimuli.

Some neurons had a similar response to the standard and to the deviant stimulus (Fig. 1A). However, many neurons had a much stronger response to the deviant than to the standard stimulus, i.e., they adapted specifically to the standard stimulus (Fig. 1B), indicating the presence of strong SSA.

### SSA quantification

**Figure 2:**
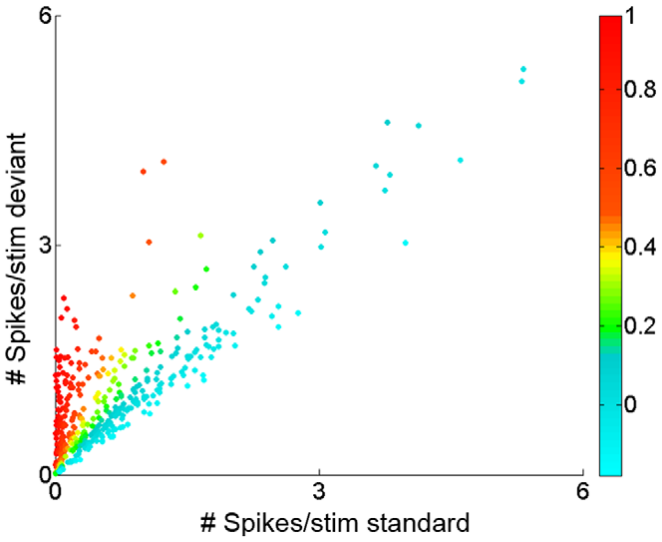
Responses of MGB neurons to the deviant and standard tones across the CSI range. Scatterplot of the response of all neurons to the deviant tone vs response to the standard tone, with points color-coded according to the CSI value (different colors, right color bar). Low CSI values (around 0) correspond to neurons having a similar response to the standard and deviant stimuli, *i.e.*, non-adapting neurons. Higher CSI values reflect a stronger response to the deviant than to the standard stimulus, i.e., adaptation to the standard. CSI values close to +1 (red color) indicate near-complete cessation of the responses to the standard tone. The most strongly responding neurons tended to be non-adapting.

In order to quantify the degree of SSA, we calculated the common SSA index (CSI) of each neuron, for each condition tested. To illustrate the relation between CSI and the responses of the neurons, we plotted the responses, elicited by the deviant versus the standard stimulus, and used different colors for the different CSI values obtained (Fig. 2). This figure shows the responses of the neurons to all conditions tested, and for this reason there are more data points than the number of neurons tested (n = 372 data points for the 93 neurons tested). For those neurons having CSI values close to 0 (blue) for a certain condition, the responses evoked by the standard were similar to those evoked by the deviant stimulus. Since the negative values found were so close to zero, we consider negative CSI values to be the result of variability in the spike counts. We took the most negative CSI value in the data set, −0.18, to represent the most extreme variance due to random fluctuations in spike counts. Using this measure, we consider the range of CSI between −0.18 and +0.18 as indicating lack of adaptation. CSI values greater than 0.18 indicate that a neuron showed a significant decrease in its response to a given stimulus when it was presented as the standard relative to the response when it was the deviant, *i.e.*, the neuron showed adaptation. CSI values close to +1 (red color) indicated near-complete cessation of the responses to the standards (Fig. 2).

### Population changes in firing rate across conditions

**Figure 3:**
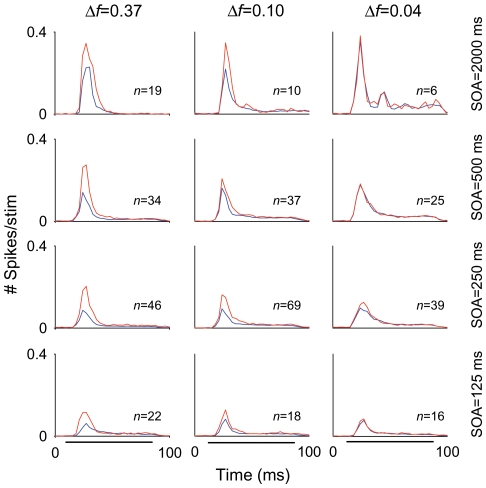
Responses of the population of MGB neurons across stimulation conditions. Averaged post-stimulus time histograms (Bin duration: 3 ms) for the entire population of MGB neurons across the different conditions tested (*Δf* and SOA) for the 90/10% probability condition. The mean firing rate elicited by both stimuli (standard, blue lines; deviant, red lines) decreased directly with SOA (SOA = 2000, 500, 250 and 125 ms; from first to fourth rows, respectively), for the different *Δfs* tested (*Δf* = 0.37, 0.10 and 0.04; from first to third columns, respectively). Numbers in each plot indicate the number of neurons for each condition. Black horizontal lines under the PSTHs of the bottom row indicate the duration of the stimulus (75 ms).

Figure 3 illustrates the average responses across the entire population of MGB neurons for the different frequency contrasts and SOAs (repetition rates) tested at the highest standard-to-deviant probability ratio (90/10%). Each plot shows the mean peristimulus time histograms of the entire population, for each combination of conditions (Fig. 3; red, deviant; blue, standard). On average, the firing rate decreased as SOA decreased, i.e., firing rate was highest at low stimulus repetition rates. This effect presumably represents a form of non-specific adaptation, which affects the responses to both standard and deviant stimuli.

The mean firing rate in response to the deviant was significantly higher than that to the standard under all conditions, except when *Δf* was 0.04 (with a small effect for SOA = 250 ms; i.e., at a repetition rate of 4/s, even at this small *Δf*). However, a population analysis of this type is biased disproportionately by neurons with high firing rates. Since the neurons with the largest responses tended to be non-adapting (e.g., compare neurons in Fig. 1A and B, tested for the same conditions; see Fig. 2 for population analysis), the responses of the highly-adapting neurons with lower firing rates are downplayed in Fig. 3.

**Figure 4:**
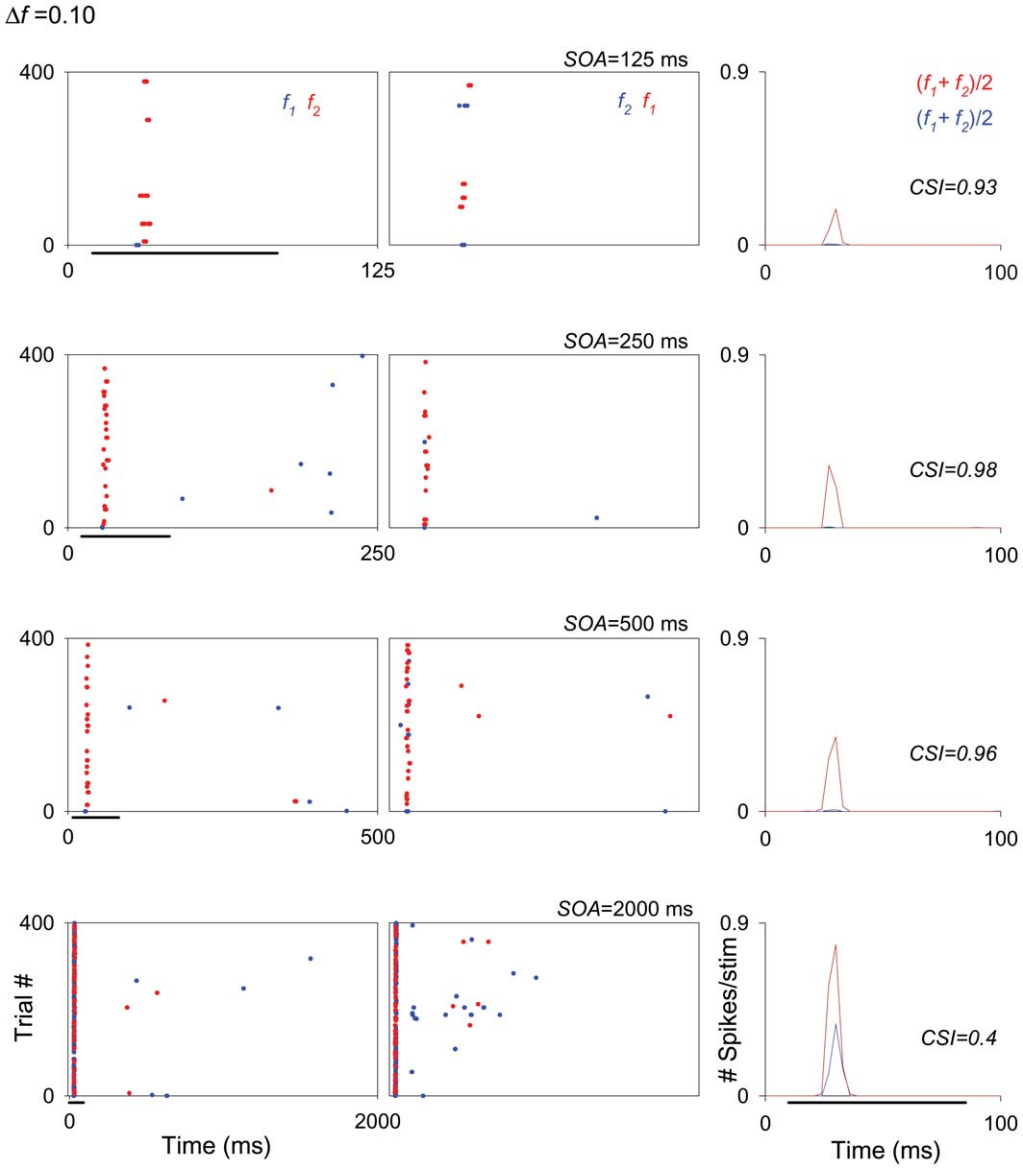
Firing rate decreases as repetition rate increases in the MGB neurons. Example of an MGM neuron showing strong SSA across different SOAs (125, 250, 500 and 2000 ms; from the first to the fourth rows, respectively) at the same *Δf* (0.10). The firing rate of this neuron decreased with decrease in SOA, it exhibited strong SSA even under extreme conditions, *i.e.*, at the combination of a *Δf* = 0.10 and SOA = 2000 ms (fourth row). In this figure and subsequent ones (*e.g*., Figs. 6, 8, 9, 11 and 13), the plots show responses as dot rasters, which plot individual spikes (red dots indicate responses to the deviant; blue dots indicate responses to the standard). Stimulus presentations are stacked along the y-axis (trial #; 400 trials each block). The time (ms) between trials (SOA) corresponds to the x-axis and is also indicated at the top right of each pair of raster plots. Because we tested different SOAs, the plots in the different rows have different x-axis scales corresponding to the SOA tested. Left and middle columns in each row represent the two blocks tested for each frequency pair (f1/f2 as standard/deviant; and f2/f1 as standard/deviant, respectively). PSTHs in the right column show the number of spikes/stimulus averaged over the two blocks [(f1+f2)/2; blue line is standard, red line is deviant]. Black horizontal lines under the plots indicate the duration of the stimulus (75 ms). The CSI calculated for each SOA condition (each row) is noted as an inset on the PSTHs.

The data collected at the shortest SOA tested (125 ms) required special treatment, since those neurons that exhibited the highest levels of SSA at higher repetition rates (SOA = 250 and/or 500 ms [4/s or 2/s]) were also those that had the largest overall reduction in their firing at high repetition rates. In the extreme case, many units (27/47) that exhibited high levels of SSA for an SOA of 250 ms completely ceased firing at the shortest SOA (125 ms) and were not included in the analysis for this condition. The remaining units (20/47) maintained some firing when tested at the 125 ms SOA condition, resulting in high CSI values for all these conditions. Figure 4 displays the responses of one of these high-SSA neurons, localized in the MGM subdivision. This neuron reduced substantially its responses for SOA = 125 ms, although its responses were not completely abolished.

### General description of SSA across the MGB population

**Figure 5:**
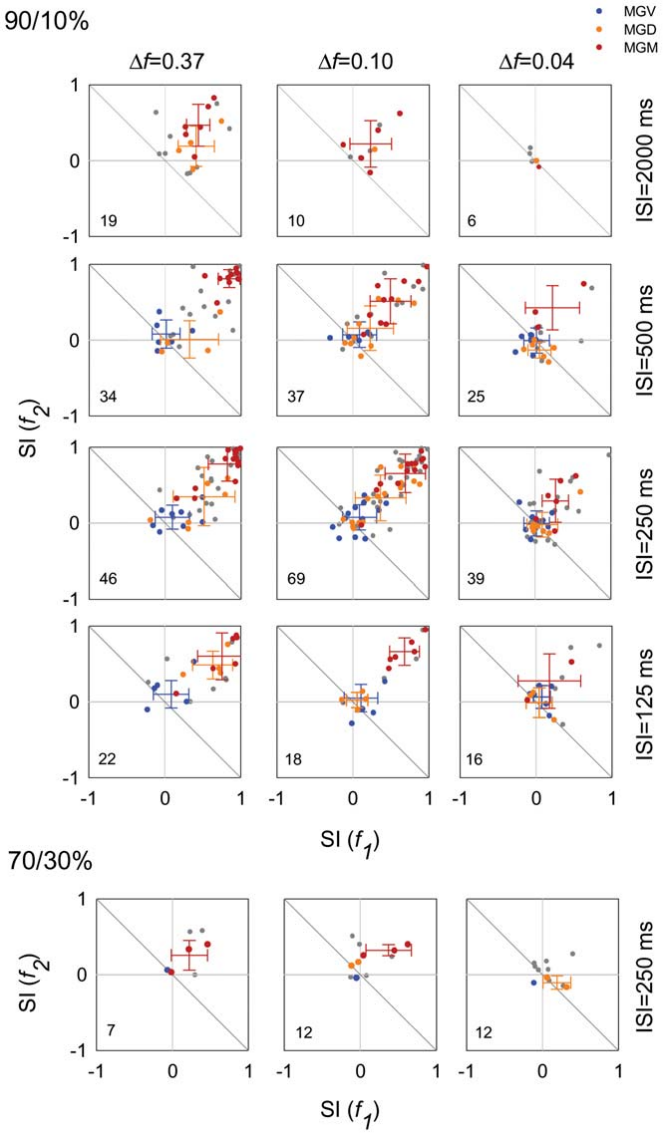
Analysis of SSA across MGB subdivisions in the population of neurons. (A, B) Scatterplots of SI(*f1*) versus (*f2*), for the different *Δfs* (0.37, 0.10 and 0.04, from first to third columns), SOAs (2000 ms, 500 ms, 250 ms, and 125 ms, from first to fourth rows) and probabilities tested (In A, 90/10%; In B, 70 30%). Each dot in each panel represents data from one neuron. Neurons that were tested for more than one set of conditions are represented in more than one panel. Numbers in the lower left quadrant of the plots represent the number of neurons tested for each condition. Blue dots represent neurons from the MGV; yellow from the MGD and red from the MGM. Grey dots represent neurons that could not be assigned with certainty to one subdivision. Crosses indicate the mean and standard deviation for the localized neurons (blue for MGV; orange for MGD; and red for MGM). For the majority of conditions SI (*fi*) values lie above the reverse diagonal indicating the presence of SSA. SSA was strongest for the intermediate SOAs (205 and 500 ms), the largest *Δfs* (0.37 and 0.10) and the 90/10% conditions. SSA was strongest in the MGM, intermediate in the MGD and weaker in the MGV subdivision.

To analyze SSA across the population of MGB neurons, we plotted the frequency-specific SSA index (SI) for the different conditions separately, and used a different color to identify the neurons that were located in the different subdivisions of MGB (Fig. 5; Red, MGM; Orange, MGD; Blue, MGV; and Gray, non-localized units).

For the majority of conditions, the plots show SI(*f_i_*) values located above the reverse diagonal, which indicates the presence of SSA [10]. The SI values for each frequency pair [SI(*f_1_*) and SI(*f_2_*)] were very similar, leading to a distribution around the diagonal in the upper right quadrant of the plot for most of the values (Fig. 5); this demonstrates that the response did not depend on the frequencies presented, but reflected genuine adaptation elicited by the standard-deviant combination.

**Figure 6:**
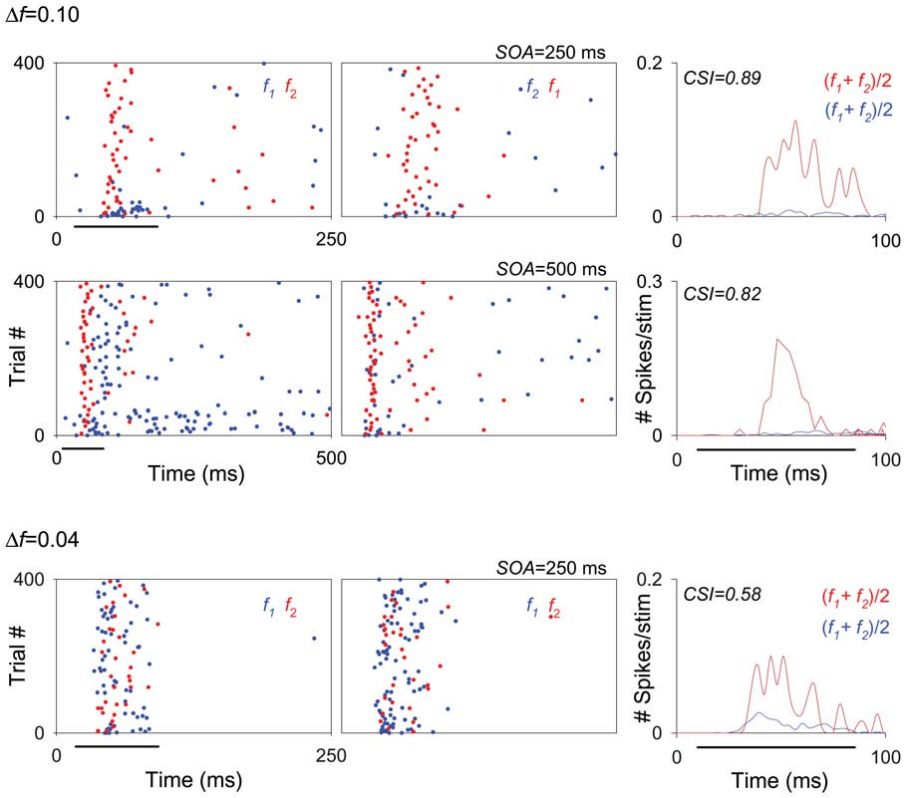
Some MGB neurons can discriminate between two very close frequencies. Example of sustained responses recorded from a neuron in the MGD, exhibiting high levels of SSA when tested at two closely spaced Δ*f*s (0.10, first and second rows; 0.04, third row). This neuron had a reduced but still high degree of adaptation for the smallest Δ*f* tested (0.04), revealing its ability to discriminate between two very close frequencies. Details of dot rasters and PSTHs are the same as in figure 4.

Across the entire population, the highest SSA values were found for the largest *Δf*s (0.37 and 0.10) at intermediate SOAs (250 and 500 ms) and the lowest deviant probability (90/10%) conditions (Fig. 5). The plots for these conditions show a cluster of points in the upper right corner (Fig. 5); these points correspond to neurons with an SI(*fi*)>0.6 for both f_1_ and f_2_, which represent the highest degree of selectivity to the rare tone. Under these conditions, a high percentage of neurons showed CSI values in the range from 0.6 to 0.99, revealing strong SSA (56% and 27% at SOA = 500, for *Δf* = 0.37 and 0.10, respectively; 46% at SOA = 250 ms for both *Δf*s; Fig. 5). Figures 4 and 6 show two examples of the responses of such neurons, localized to the MGM and MGD, respectively (For the units in Figs. 4 and 6, CSI>0.82 when *Δf*≥0.1 and SOA = 250 or 500 ms). At the shortest SOA (125 ms), some neurons failed to respond at all and for this reason fewer neurons are represented in these plots (Fig. 5). Even so, some neurons maintained a high degree of SSA under this condition (Fig. 4, first row; *Δf* = 0.1 and SOA = 125; CSI = 0.93).

Although the amount of SSA was reduced for the largest SOA (2000 ms) and the smallest *Δf* (0.04), we recorded neurons that exhibited robust SSA under each of these conditions (Fig. 5). Figure 4 shows the responses of a neuron that had a reduced, but still high degree of adaptation at an SOA of 2000 ms (fourth row: *Δf* = 0.10, CSI = 0.40; compared to CSI>0.9 for shorter SOAs). Other neurons had CSI>0.6 at SOAs of 2000 ms. For the smallest *Δf* (0.04) some neurons had CSI>0.6 as well (for SOA = 250 ms and 500 ms, respectively; Fig. 5). Figure 6 shows an example of a neuron that had a reduced, but still high degree of adaptation with *Δf* = 0.04 (SOA = 250 ms, CSI = 0.58). However, no neurons showed SSA when tested with the combination of *Δf* = 0.04 and SOA = 2000 ms. This was in fact the only combination out of the 15 tested that did not elicit any SSA in the MGB population (Fig. 5).

The amount of SSA was lower for the 70/30% condition; although some neurons exhibited high SI values under this condition, they were lower than for the 90/10% condition at the same SOA and *Δf* s (Fig. 5; compare row 3 and row 5).

### SSA across MGB subdivisions

**Figure 7:**
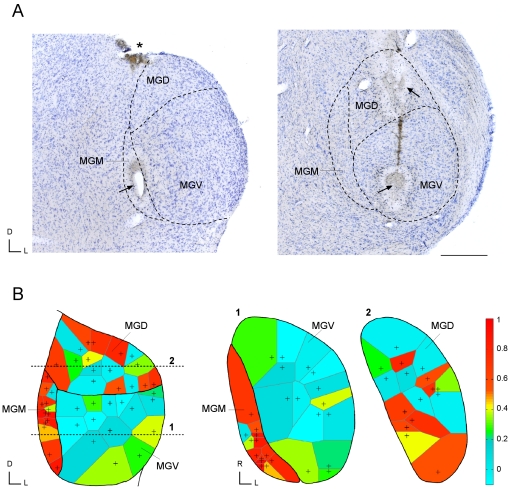
Location of recorded neurons and topographical organization of SSA across the MGB. (A) Nissl stained sections showing the MGB in the transverse plane. On the left (caudal), arrows indicate the electrolytic lesion in the MGM marking the recording site of the neuron shown in figure 9. Asterisk indicates another lesion for reference. On the right (rostral), arrows indicate an electrolytic lesion in the MGD and another one in the MGV, marking the recording site of the neuron shown in figure 8. Asterisk shows the recording track. D, dorsal; L, lateral; Calibration bar  = 500 µm. (B) Topographical organization of SSA within the MGB subdivisions, for the *Δf* = 0.10 at SOA = 500 ms condition. The center of tessellated polygons in the maps represents the sites at which the neurons were recorded. Each polygon was colored according to the CSI value of the neuron recorded at that site. The bar on the right represents the color scale used for the CSI range. Both the transverse projection (on left) and the horizontal projections through the MGV/MGM (section 1) and MGD (section 2) show that SSA was strongest throughout the entire MGM followed by the caudal, medial and dorsal regions of the MGD. SSA was very weak in the center of the MGV, but somewhat greater in its periphery.

Figure 5 clearly indicates that SSA was stronger in the medial subdivision (red dots), than in the other subdivisions. SSA was smallest in the ventral subdivision (blue dots) and intermediate in the dorsal subdivision (orange dots). Figure 7A shows Nissl stained-sections of the MGB with marked electrolytic lesions, corresponding to the location of neurons recorded in the different MGB subdivisions. To analyze the topographical organization of SSA within the MGB subdivisions, we constructed Voronoi tessellations [36] for the combination of conditions for which the sample was largest (*Δf* = 0.10 at SOA = 500 ms; Fig. 7B), using a color scale for the CSI values as in Figure 2. This analysis confirmed that SSA was strongest throughout the entire medial subdivision (MGM) followed by the caudal, medial and dorsal regions of the dorsal subdivision (MGD) (Fig. 7B). SSA was weaker in the ventral subdivision (MGV), being essentially non-existent in the center and somewhat greater in the periphery (Fig. 7B).

To quantify these observations, we performed a 3-Way ANOVA to look for significant effects and interactions between subdivisions and the conditions tested, for the 90/10% probability condition. The analysis of CSI across subdivisions (factors: subdivision x *Δf* x SOA) indicated that all the factors had significant effects on the CSI [*F*_(2,211)_ = 89.6, *F*_(2,211)_ = 28.4 and *F*_(3,211)_ = 7.6, respectively; *p*<0.001 in all cases]. *Post hoc* comparisons (Tukey's HSD, *p*<0.05) confirmed that the average CSI in the MGV (0.01) was not different from zero and was significantly smaller than that in the MGM and in the MGD (0.52 and 0.17, respectively); SSA in the MGM was significantly larger than in the MGD as well. CSI was significantly dependent on *Δf*, and decreased monotonically with *Δf* (mean CSI 0.39, 0.25 and 0.07 for *Δf = *0.37, *Δf* = 0.1 and *Δf* = 0.04, respectively; all significantly larger than 0, and all pairwise differences significant). SSA depended significantly on the SOA and was smaller for the largest SOA (2000 ms; mean 0.06) than for the other SOAs (0.25, 0.34 and 0.29 for SOA = 500 ms, 250 ms and 125 ms respectively); SSA levels at the three faster rates were not significantly different from each other.

Next we tested for interactions between the different factors. In this analysis the data corresponding to SOA = 2000 ms were removed because this condition was not tested in the MGV subdivision at all. The main effects of subdivision and *Δf* were similar to the previous analysis (both the order and the significance of the pairwise comparisons were preserved in the presence of possible interactions). SOA had a non-significant main effect [*F*_(2,182)_ = 2.6; *p* = 0.08]. This was not surprising given the finding that only the SOA = 2000 ms condition had significantly different SSA from the other SOAs. The only significant interaction was between subdivision and *Δf* [*F*_(4,182)_ = 3.97; *p*<0.01]. *Post hoc* comparisons (Tukey's HSD) revealed that in the MGV the average CSI at all *Δf*s were not significantly different from 0 and from each other (0.11, 0.07 and 0.02 for *Δf* = 0.37, 0.1 and 0.04 respectively); In the MGD the mean CSI at *Δf* = 0.04 was not significantly different from 0 (0.005), but at the other *Δf*s was larger than 0 (0.36 and 0.23 for *Δf* = 0.37 and 0.10, respectively); in the MGM the mean CSI at all three *Δf*s were significantly larger than 0 (0.79, 0.61 and 0.29 for *Δf* = 0.37, 0.1 and 0.04 respectively). Furthermore, in both MGD and MGM, the effects at *Δf* = 0.04 were significantly smaller than at the other two *Δf*s; the effect at *Δf* = 0.37 was larger than at *Δf* = 0.1, but the two were not significantly different from each other in any subdivision.

### SSA within MGB subdivisions

The previous analysis is conservative, as it did not account for a possible effect of neuron on *Δf* and SOA: CSI values for the same neuron under different conditions tended to be correlated. To analyze the effect of neuron on SSA we performed an N-Way ANOVA with a nested design (introducing units within subdivisions). This analysis demonstrated a strong effect of neuron on the variation of SSA (*F*_(73,138)_ = 5.26; *p*<0.0001).

To look at the effects of neuron, *Δf*, and SOA on SSA within subdivisions, we performed 3-Way ANOVAs for each subdivision separately, with the neurons as a random factor. This analysis demonstrated a significant effect of neurons in each subdivision separately [*F*_(21,39)_ = 2.75; *p*<0.01; *F*_(28,34)_ = 14.92; *p*<0.001; and *F*_(24,56)_ = 3.10; *p*<0.001, for the MGV, MGD and MGM respectively]. Within subdivisions, after controlling for neuron, the effect of *Δf* was not significant in the ventral and dorsal subdivisions [*F*_(2,21)_ = 2.06; *p* = 0.1 and *F*_(2,34)_ = 1.69; *p* = 0.2, respectively], but was significant in the medial subdivision [*F*_(2,56)_ = 5.06; *p*<0.01]. These results are consistent with the analysis of the interactions between *Δf* and subdivision presented above, which showed that the effect of *Δf* was more pronounced in the MGM, weaker in the MGD and absent in the MGV. Most importantly, the analysis within subdivisions reveals a strong effect of SOA. CSI had a monotonically-inverse relationship with SOA in all three subdivions [*F*_(2,39)_ = 5.49; *p*<0.01; *F*_(3,34)_ = 21.78; *p*<0.001; and *F*_(3,56)_ = 10.47; *p*<0.001, for the MGV, MGD and MGM respectively].

**Figure 8:**
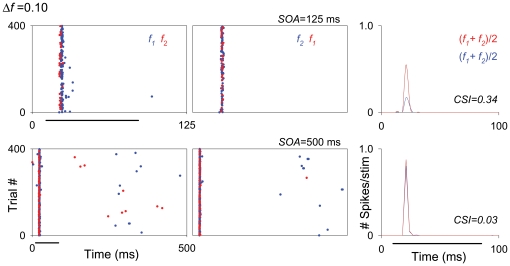
High repetition rates and large *Δf*s can elicit SSA in some MGV neurons. Example of an onset neuron from the MGV tested at two different SOAs (500 and 125 ms) for the same *Δf* (0.37). This neuron did not show SSA for the longest SOA tested (500 ms, second row) but did show some adaptation at the shortest SOA (125 ms, first row). Details of dot rasters and PSTHs are the same as in figure 4. The location of this neuron is shown in figure 7A.

*Post hoc* comparisons showed that in the MGV, SSA at the shortest SOA (125 ms) was significantly larger than at SOA = 250 ms; the other comparisons were not significant. Figure 8 shows an example of a neuron, localized to the MGV, that exhibited SSA at SOA = 125 (CSI = 0.34; first row) but not at SOA = 250 ms (CSI = 0.03; not shown) or SOA = 500 ms (CSI = 0.03; second row).

In the MGD, as in the MGV, SSA at SOA of 125 ms was significantly larger than at 250 ms. In the MGM, on the other hand, there was no significant difference between SOAs of 125, 250 and 500 ms. Figure 4 shows an example of a neuron, localized to the MGM, that exhibited the same degree of adaptation for all of these different SOAs (first, second and third rows; 125, 250 and 500 ms, respectively).

**Figure 9:**
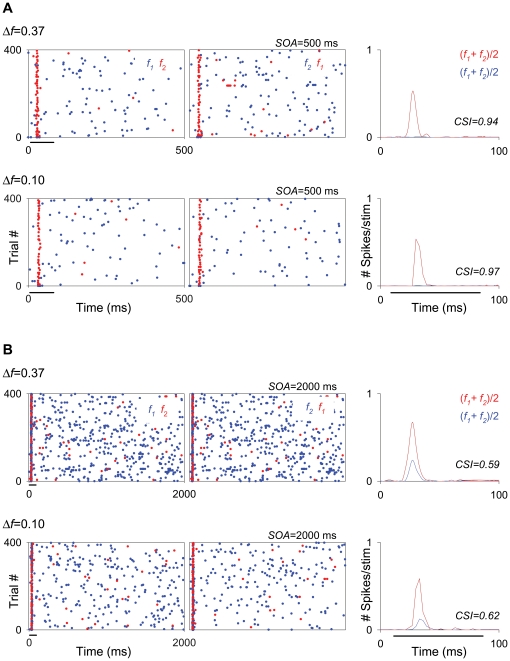
Low repetition rates elicit high SSA in some MGM neurons. Example of an onset neuron with spontaneous activity recorded in the MGM, showing strong adaptation under all of the conditions tested. (A) the neuron exhibited extreme adaptation when tested at the same SOA (500 ms) for two different *Δfs* (0.37 and 0.10; first and second rows, respectively). (B) the neuron showed somewhat lower adaptation when tested at the longest SOA (2000 ms; same *Δfs* as in A). In both A and B, the adaptation was similar for both *Δf* conditions. Details of dot rasters and PSTHs are the same as in figure 4. The location of this neuron is shown in figure 7A.

In the MGD and the MGM, SSA at the longest SOA (2000 ms) was significantly reduced with respect to the other SOAs. Figure 9 shows an example of a neuron, localized to the MGM, that showed reduced CSI at the longest SOA (second row in A and B) compared to shorter SOAs (first row in A and B), for two different *Δf*s (0.37 and 0.10).

### Time course of SSA

**Figure 10:**
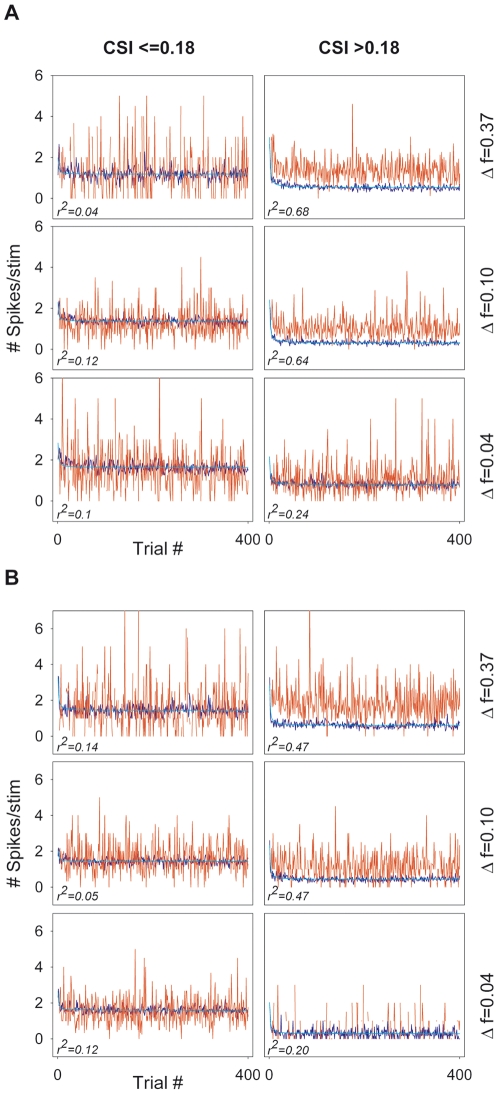
Time course of adaptation in the population of MGB neurons. Average population firing rate (spikes/stimulus) versus trial number for SOA = 250 ms (A) and SOA = 500 ms (B) and the different *Δfs* tested, indicated to the right of each row. In both A and B, the left columns correspond to non-adapting neurons (CSI≤0.18) and right columns to adapting neurons (CSI>0.18). The response of the adapting neurons to the standard stimulus strongly declined after the first trials. A high proportion of their adaptation to the stimulus was explained by a polynomial inverse regression model [*f*  = *y0*+(*a*/*x*)], for the majority of conditions; the amount of variance explained was reduced for the smallest *Δf* (0.04) (*r*2 = 0.24, 0.20 in A and B, respectively; *p* <0.001 for both conditions) and was very low for the non-adapting neurons, under all conditions.

In order to study the dynamics of SSA in the population of MGB neurons, we calculated the average population firing rate versus trial number, for the two SOA conditions that showed the highest levels of adaptation (SOA = 250 and 500 ms), and for which we collected the most data (Figure 10). We analyzed separately the non-adapting neurons (CSI≤0.18) and the adapting neurons (CSI>0.18) (Fig. 10 A and B; left and right columns, respectively). In the initial trials, the average responses to the standard and deviant stimuli were similar (Fig. 10). The adapting neurons maintained or only slightly reduced their response to the deviant stimulus through the trials, while the response to the standard declined more strongly after the first few trials (Figs. 10; right column). The responses of non-adapting neurons to the standard also showed some decrement after the first trials, especially for the *Δf* = 0.37 conditions (Fig. 10; left column), but this decrement was much smaller than that of the adapting neurons. As a consequence, non-adapting neurons maintained a higher firing rate to the standard than the adapting neurons across the trials, for all conditions (Fig. 10). We then fitted the responses to the standard with an exponential decay regression model [*f* = a*exp(-b*x)] and a polynomial inverse first order regression model [*f* = *y_0_*+(*a*/*x*)]. The polynomial inverse first order model was the one that provided the best fit to the responses to the standard across trials, for all conditions. A high proportion of the adaptation to the standard by the adapting neurons was explained by this model, for the majority of conditions (SOA = 250 ms: *r* ^2^ = 0.68 and 0.64, at *Δf* = 0.37 and 0.10, respectively; SOA = 500 ms: *r^2^* = 0.47 at both *Δf*s; *p*<0.001 for all conditions; Fig. 10A and B, respectively; right column, first and second rows). For the smallest *Δf* (0.04) the variance explained by the model was reduced (*r* ^2^ = 0.24, 0.20 at SOA = 250 and 500 ms, respectively; *p*<0.001 for both conditions; Fig. 10, right column, third row). For the non-adapting neurons, the variance explained by the model was very low for all conditions (SOA = 250 ms: *r^2^ = *0.04, 0.12 and 0.1, for *Δf* = 0.37, 0.1 and 0.04, respectively; SOA = 500 ms: *r^2^* = 0.14, 0.05 and 0.12, for *Δf* = 0.37, 0.1 and 0.04, respectively; *p*<0.001 for all conditions; Fig. 10, left column). The smaller amount of variance explained by the regression model in these conditions presumably reflects the minor amount of adaptation of the non-adapting neurons.

### SSA in relation to discharge patterns and latencies

**Figure 11:**
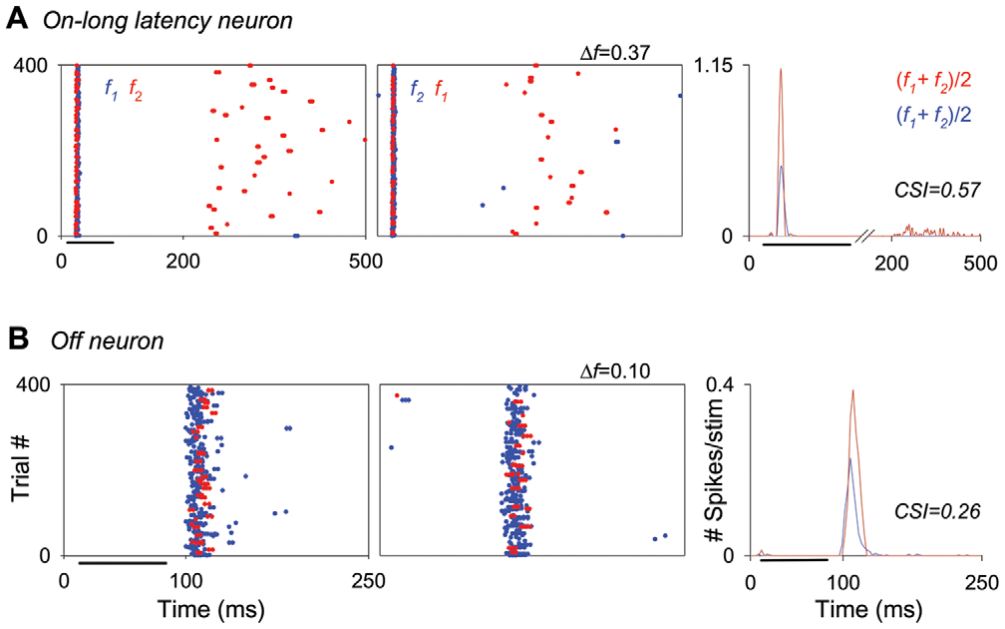
Some MGB neurons with on-late and off response types show adaptation. (A) Example of an on-late neuron in the MGD. This neuron responded with a brief onset burst at a relatively short latency (14.8±0.4 and 16±0.5 ms; average of the mean first-spike latency for f1 and f2 when deviant and standard, respectively) followed by a long-duration burst at a much longer latency (245.8±7 ms; average of the mean first-spike latency for f1 and f2 when deviant). The neuron showed some adaptation in the onset burst but much stronger adaptation in the late burst (CSI = 0.31 and 0.98, respectively; CSI = 0.57 for the entire response time window). Details of dot rasters and PSTHs are the same as in figure 4. (B) Example of an offset neuron from the MGV that exhibited some adaptation (CSI = 0.26). Details of dot rasters and PSTHs are the same as in figure 4.

Over half of the neurons that we recorded from the MGB had onset responses to auditory stimuli (53%; 49/93, *e.g.*, Figs. 4, 8 and 9) while 24% had sustained responses (22/93, *e.g*., Fig. 6; defined as neurons that responded for 50 ms or more [23], [25], in response to a 75 ms stimulus). In addition, some units (10%; 9/93) had two different response components: a brief onset burst at a relatively short latency (10–30 ms) followed by a long-duration burst at a much longer latency (>115 ms). Both response components were tuned to the same frequency range, but were clearly segregated in time. We refer to these units as on-late units. Fig. 11A shows an example of one of these units, recorded from the MGD and exhibiting SSA. A small percentage of neurons had offset responses (8%; 7/93). Figure 11B shows an example of one of these units, recorded from the MGV, also exhibiting SSA. Finally, some units (2%; 2/93) had on-off responses.

A high percentage of the units recorded (56%; 52/93) exhibited SSA for at least one of the two largest *Δf*s (0.37 and 0.10) at one of the intermediate SOAs (250 and 500 ms). Of these units, the majority were onset responders (73%; 38/52), followed by on-late responders (12%; 6/52), and a minority of sustained, long latency and offset responders (6, 6 and 4%; 3, 3 and 2/52, respectively). Of the non-adapting neurons (44%; 41/93), the majority were sustained responders (44%; 18/41), followed by onset responders (24%; 10/41), and by smaller percentages of offset, on-late, long latency and on-off responders (12, 7.3, 7.3, 5%; 5, 3, 3, 2/41, respectively). The distributions of unit types among the adapting and non-adapting neurons was significantly different (χ^2^ = 22, df = 2, *p*<0.001).

**Figure 12:**
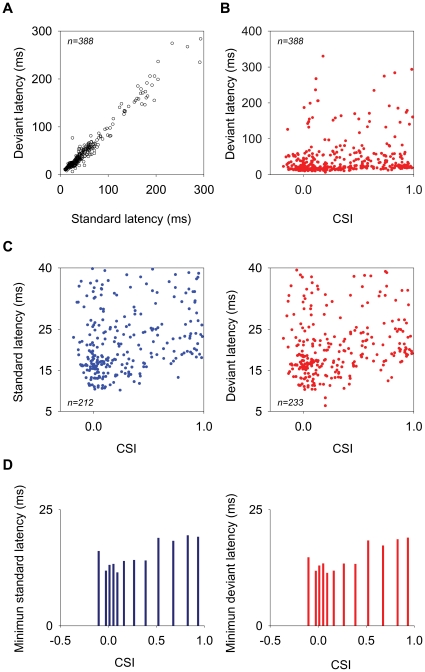
Response latencies in the MGB population of neurons. (A) Mean first-spike latencies to the deviant versus standard stimulus for the MGB population. Latencies to the deviant were on average significantly shorter than those to the standard stimulus (Mean = 42.9 and 45.7 ms, respectively; paired *t-test*: t = 5.79, n = 388, d.f = 387, p<0.001). (B) Mean first-spike latencies to the deviant versus CSI. The shortest latencies o f highly adapting neurons were longer than those of non-adapting neurons. (C) Short-latency responses (<40 ms) to standard (left plot) and deviant (right plot) versus CSI. (D) The 10th percentile of the minimum latency distribution for the standard (left plot) and deviant (right plot) at different ranges of CSI. The minimal latencies of neurons with high CSI values (>0.5) were longer than those with lower CSI values, except for the most negative CSI values.

**Figure 13:**
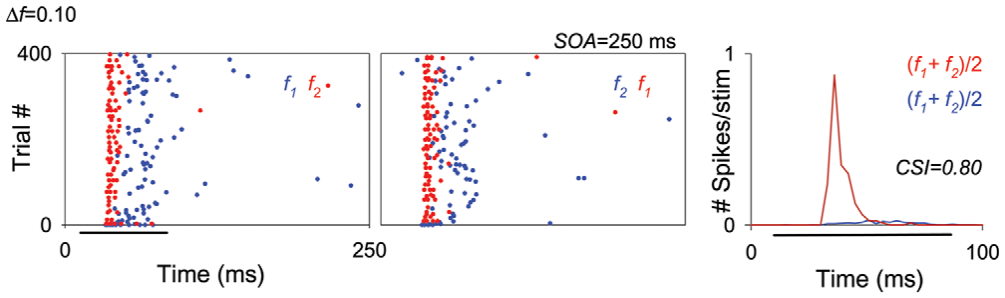
MGB neurons show shorter latencies to the deviant than to the standard stimulus. Example of an adapting neuron that responded with a much shorter latency to the deviant than to the standard stimulus (26±0.4 and 41.1±1.6 ms; average of the mean first-spike latency between f1 and f2 when deviant and standard, respectively), for a *Δf*  = 0.10 at SOA = 250 ms condition. The latency of the response to the first stimulus presentation of the set was similar for both stimuli, it was even slightly shorter to the standard (24.5±0.7 and 23.5±0.2 ms, average of the first-spike latency to the first stimulus presentation between f1 and f2, when deviant and standard, respectively). Details of dot rasters and PSTHs are the same as in figure 4.

To analyze the relationship between SSA and response latency, we plotted the mean first-spike latencies to the standard and deviant stimuli for all neurons under all conditions tested to determine whether their means differed (Fig. 12A). For the on-late neurons, we calculated separately the latency of their onset component (<115 ms) and the latency of their long latency component (>115 ms). For this reason, 16 out of the 388 data points in figure 12A correspond to the long latency components of these neurons. The latency of the responses evoked by the deviant was significantly shorter on average than that evoked by the standard (42.9 and 45.7 ms, respectively; paired *t-test*: t = 5.79, d.f. = 387, p<0.001). Figure 13 shows an example of a neuron that exhibited a much shorter latency to the deviant than to the standard stimulus. For this neuron, the mean first spike latency of the response to the deviant was on average 15.1 ms shorter than that to the standard stimulus for an SOA = 250 (26±0.4 and 41.1±1.6 ms; average of the mean first-spike latency for f_1_ and f_2_ when deviant and standard, respectively; Fig. 13); and 7.6 ms shorter for an SOA = 500 ms (24.7±1.1 and 32.3±1.2 ms; average of the mean first-spike latency for f_1_ and f_2_ when deviant and standard, respectively; not shown). As would be expected, for this same neuron, the latency of the response to the first stimulus presentation of the set was similar for both stimuli (24±0.7 and 23.5±0.3 ms, average of the first-spike latency to the first stimulus presentation for all stimuli, at SOA = 250 and 500 ms, respectively).

Figure 12A shows that the latencies of the MGB neurons were distributed over a broad range; to determine whether the latency of the neurons was related to the amount of adaptation that they exhibited, we plotted their mean first-spike latencies to the deviant versus their CSI (Fig. 12B). While most latencies were distributed similarly across all values of CSI (Fig. 12B), the shortest latencies of neurons with large CSIs tended to be somewhat longer than the shortest latencies of those neurons with small CSIs. To better visualize this issue, Fig. 12C displays only short-latency responses (<40 ms) to standards (left plot) and deviants (right plot) as a function of CSI. Although there was considerable overlap at all CSIs, the CSI did have a significant effect on the variation of the minimal latencies (<25 ms) for both standard and deviant stimuli [one-way ANOVA grouped by CSI: *F*_(11,171)_ = 3.64; *p*<0.001; and *F*_(11,203)_ = 4.72; *p*<0.001, for deviant and standard respectively]. The minimal latencies of neurons exhibiting high CSI values (>0.5) were longer than those exhibiting lower CSI values, except for the most negative CSI values (Fig. 12E displays the 10% percentile of the minimum latency distribution at different ranges of CSI; standard, left plot; deviant, right plot).

## Discussion

In this study, we demonstrated that a significant percentage of neurons in the MGB show adaptation of their responses to tones in a stimulus-specific manner. Neurons in the MGM exhibited the strongest adaptation followed by neurons in the MGD. On the other hand, SSA was absent in the MGV under most of the tested conditions, and was found there only for the shortest SOAs used here (125–250 ms). Thus, SSA is prominent in the non-lemniscal divisions of the auditory thalamus, but weak in the ventral, lemniscal, division.

The stimulation conditions that evoked the strongest SSA in MGB neurons were the two largest *Δfs* (0.37, 0.10) at the intermediate SOAs (250 and 500 ms). Under these conditions a high percentage of MGM and MGD neurons showed strong to extreme adaptation. Moreover, some neurons in these subdivisions showed SSA even when tested with the longest SOA (2000 ms) as well as the smallest *Δf* (0.04). Our study thus revealed the ability of these neurons to discriminate between two very close frequencies, both of which are well within their frequency response area. Such hyperacuity was demonstrated before in cat AC [10] and rat IC [4]. Our results together with those of others [1], [4], [10], [12], [13] demonstrate the ubiquity of SSA in neurons throughout the auditory system, from the midbrain up to the auditory cortex.

### Comparison with previous studies

Two recent studies tested SSA in MGB. Yu and colleagues [13] studied SSA in the rat MGB and thalamic reticular nucleus, a subdivision of the thalamus that lies outside the MGB. They demonstrated strong SSA in the thalamic reticular nucleus and weaker SSA in MGB. Anderson and colleagues [1] reported SSA in mouse MGB, but tested fewer conditions and showed weaker SSA than reported in the current study. We demonstrated that some neurons in the rat MGB exhibit very strong SSA even under the most extreme conditions tested (SOAs = 2000 ms or Δf = 0.04). In fact, we found a few neurons in MGB with CSI values as high as those reported by Yu et al. [13] in the thalamic reticular nucleus, even when using SOAs twice as long.

The parameter ranges over which SSA occurs in the rat MGB are similar to those in cat AC [10] and rat IC [4]. SSA in the rat IC was tested only for relatively short SOAs (up to 500 ms), so we cannot compare the IC with the MGB for the largest SOA. However, our MGB results (SOA = 2000 ms, inter-tone duration>1900 ms) can be compared with those from the cat AC study (SOA = 2000 ms, inter-tone duration>1700 ms). A SOA of 2000 ms corresponds to the most extreme condition for which Ulanovsky and colleagues [10] showed single units exhibiting SSA in A1 (CSI≈0.3). We found higher values of SSA for this condition in the MGB (up to CSI = 0.7) than the previous study in the AC, but only outside the MGV. The MGV receives input from the central nucleus of the IC and is the main source of ascending input to A1 [28]–[31]. In this context, it is worth mentioning that SSA found in the central nucleus of the IC [4] is only relatively large for the shortest SOA tested (125 ms). Thus, the MGB and IC data tightly links SSA in subcortical regions to the non-lemniscal pathway [4], [6].

CSI values reported in A1 of the cat are far in excess of the values measured in cat MGB, presumably in the ventral division [10], or in rat MGV. Thus, our results suggest that A1 is the first lemniscal station in which SSA is widespread and strong. SSA in A1 may therefore express the combined result of the rather weak SSA found in MGV augmented by intracortical mechanisms [37] and possibly by the weaker (but still present) non-lemniscal input to A1, either directly from the MGM [38] or indirectly through feedback connections from higher auditory areas. For example, a recent study [39] has demonstrated that reversible thermal deactivation of AAF alters A1 responses but AAF responses are not altered by A1 deactivation. These authors suggest a unidirectional flow of information from the non-lemniscal to the lemniscal pathway. If so, the SSA observed in A1 may be modulated by the influence of AAF [10].

Strongly adapting neurons in both the MGB and the IC [4], [6] were mainly onset responders, with relatively short latencies (<40 ms) for both the standard and the deviant stimuli. Nevertheless, the shortest latencies of the neurons that showed strong adaptation for a certain condition in the MGB were significantly longer than those of weakly adapting neurons for a certain condition, to both the deviant and standard stimuli. These slightly longer latencies could simply reflect lower firing rates of the neurons showing strong adaptation for a given condition, as the mean first-spike latency can be affected by the response strength, or it could be due to additional neuronal processing, for example cortical modulation of these neurons [10]. This hypothesis needs to be addressed in future experiments, *e.g*., by reversibly inactivating the AC [40], [41].

### SSA and sensory memory

A neuron exhibiting SSA integrates information about recent stimulus history in order to respond more strongly to a rare stimulus. SSA therefore embodies a short-term memory trace that determines the response of the neuron to subsequent stimulation [7], [42], [43]. We demonstrated that a polynomial scale-invariant model explained a high proportion of MGB neurons' adaptation to the standard stimulus. Such a power law model may indicate that adaptation occurs over a range of time-scales [44], [45], so that in contrast to exponential adaptation, activity more than a few time constants back, although deemphasized, is not discarded. Indeed, SSA in A1 neurons appears to occur on several time scales concurrently, spanning many orders of magnitude, from hundreds of milliseconds to tens of seconds [11], paralleling the behaviour of large neuronal populations as recorded in human event-related potentials [46]. SSA was therefore proposed as a candidate neuronal mechanism for auditory sensory memory and deviance detection as reported in human MMN studies [5], [47], [48]. However, the MMN component occurs at 100–250 ms after the onset of an acoustic change, while SSA occurs at much shorter latencies [4], [11]. Indeed, a recent study based on neuronal recordings and evoked local field potentials (eLFP) in the awake rat found enhanced responses to deviants in eLFP but did not find the late deviant response component that would have been the equivalent to the human MMN [12]. Thus, SSA has been suggested to lie upstream of MMN generation. Recent studies in humans demonstrated that deviance detection can take place as early as 30 ms after stimulus onset, suggesting that early change detection processes occur upstream of MMN generation [49], [50]. As in the animal models that have been studied, deviance detection in humans occurs at multiple levels in the auditory pathway, from the brainstem up to higher-order cortical areas [49], [50].

Here, we demonstrate that the latencies of strongly adapting neurons in the MGB span a range between approximately 10 ms to 250 ms [51], covering the range of the different components of the MMN in humans [47], [49], [50] and rats [52], [53]. The majority of the strongly adapting neurons in the MGM subdivision had onset responses with short latencies. These neurons could be participants in a bottom-up stream of SSA [51]. However, some strongly adapting neurons in the MGB had very long onset latencies (>150 ms) and some neurons had two different components in their response, *i.e*., a short latency component (<40 ms) together with a long latency one (>150 ms). The timing of the long latency components of these neurons is similar to the range of the latencies of the MMN component of human ERPs (≈200 ms; [14], [47], [54]). This suggests that there might be some relationship between the SSA exhibited by this population of neurons and the MMN component. Our data and those of others who found evidence of MMN subcortically (reviewed in [19]) indicate that MMN may be generated by processes that include both bottom-up processing and corticothalamic feedback loops.

The presence of strong SSA in the auditory thalamus suggests that SSA is important for the type of processing performed there. For example, the very strong SSA found in the MGM is consistent with its role as a major auditory input to the fear circuit in the amygdala [24], [55]. The role of SSA expressed in IC and MGB in shaping SSA in A1 is less clear, and it may well be that SSA in A1 is generated at least in part *de-novo*. Nevertheless, the presence of strongly-adapting neurons in non-lemniscal divisions of the MGB may indicate the active role of these neurons in the generation, transformation or modulation of SSA expressed in other parts of the auditory system. Testing such a role would require future work.

## Materials and Methods

### Surgical procedures

Experiments were performed on 21 adult rats with body weights between 150–250 g. All experiments were carried out at the University of Salamanca with the approval of, and using methods conforming to the standards of, the University of Salamanca Animal Care Committee.

Surgical anaesthesia was induced and maintained with urethane (1.5 g/kg, i.p.), with supplementary doses (0.5 g/kg, i.p.) given as needed. Urethane was selected as an anaesthetic because its effects on multiple aspects of neural activity, including inhibition and spontaneous firing, are known to be less than those of barbiturates and other anaesthetic drugs (e.g. [56]). The trachea was cannulated, and atropine sulphate (0.05 mg/kg, s.c.) was administered to reduce bronchial secretions. Body temperature was maintained at 38°C±1°C. Details of surgical preparation were as described elsewhere [57], [58]. The animal was placed in a stereotaxic frame in which the ear bars were replaced by hollow specula that accommodated a sound delivery system.

### Acoustic stimuli and electrophysiological recording

A craniotomy was performed to expose the cerebral cortex overlying the MGB. A tungsten electrode (1–2 MΩ; [59]) was lowered through the cortex and used to record extracellular single unit responses. Neuron localization in the MGB was based on stereotaxic coordinates, physiological criteria of tonotopicity and response properties [23]–[25]. Subsequent histological verification was performed using electrolytic lesions (5–10 µA for 5–10 s) to mark recording sites [60].

Stimuli were delivered through a sealed acoustic system [61], [62] using two electrostatic loudspeakers (TDT- EC1) driven by two TDT-ED1 modules. Pure tone bursts were delivered to the contralateral ear under computer control using TDT System 2 (Tucker-Davis Technologies) hardware and custom software [4], [63], [64]. The output of the system at each ear was calibrated in situ using a ¼’’ condenser microphone (Brüel and Kjær 4136, Nærum, Denmark) and a DI-2200 spectrum analyser (Diagnostic Instruments Ltd., Livingston, Scotland, UK). The maximum output of the TDT system was flat from 0.3–5 kHz (∼100±7 dB SPL) and from 5–40 kHz (90±5 dB SPL). The highest frequency produced by this system was limited to 40 kHz. The second and third harmonic components in the signal were 40 dB or more below the level of the fundamental at the highest output level [60], [65].

Tones were 75 ms duration with a 5 ms rise/fall time. The electrode was advanced using a Burleigh microdrive. Action potentials were recorded with a BIOAMP amplifier (TDT), the 10X output of which was further amplified and bandpass-filtered (TDT PC1; f_c_, 500 Hz and 3 kHz) before passing through a spike discriminator (TDT SD1). Spike times were logged on a computer by feeding the output of the spike discriminator into an event timer (TDT ET1) synchronized to a timing generator (TDT TG6). Stimulus generation and on-line data visualization were controlled with custom software. Spike times were displayed as dot rasters ordered by the acoustic parameter varied during testing. Search stimuli were pure tones or noise bursts.

To the extent possible, the approximate frequency tuning of the cell was audiovisually determined. The minimum threshold and best frequency (BF) of the cell were obtained by an automated procedure with 2–5 stimulus repetitions at each frequency and intensity step. The monaural frequency response area (FRA, e.g., Fig. 1), i.e., the combination of frequencies and intensities capable of evoking a response, was then obtained automatically using a randomized stimulus presentation paradigm and plotted using EXCEL, SIGMAPLOT and MATLAB software. The stimuli used to generate FRAs in single units were pure tones with a duration of 75 ms. Frequency and intensity of the stimulus were varied randomly (0–100 dB attenuation in 5 or 10 dB steps and in 25 frequency steps from 0.1–40 to cover approximately 2–3 octaves above and below the BF; [60], [65]).

### Stimulus presentation paradigms

For all neurons, stimuli were presented in an oddball paradigm similar to that used to record mismatch negativity responses in human studies [54] and more recently in the cat auditory cortex [10], [11] and rat inferior colliculus [4]. Briefly, we presented trains of two different pure tone stimuli (f_1_ and f_2_), at a level of 10–40 dB above threshold. Both frequencies were within the excitatory frequency response area previously determined for the neuron (Fig. 1). We presented a train of 400 stimuli containing both frequencies in a pseudo-random order at a specific repetition rate. One frequency (f_1_) was presented as the standard (i.e., high probability within the sequence); interspersed randomly with the second frequency (f_2_) presented as the deviant (i.e., low probability within the sequence). Special care was taken to choose a frequency pair that elicited similar spike counts when presented individually, to ensure that all differences in response were solely due to the statistics of the stimulus ensemble (Fig. 1). The custom software allowed us to independently vary the probability of the deviant stimulus and the amount by which its frequency varied from that of the standard. After obtaining one data set, the relative probabilities of the two stimuli were reversed, with f_2_ as the standard and f_1_ as the deviant (total number of stimuli for the frequency pair, 800).

The same paradigm was repeated varying the probability of the standard/deviant stimuli (90/10% and 70/30%), the stimulus onset asynchrony (SOA = 2000 ms, 500 ms, 250 ms, and 125 ms), and the frequency contrast between the standard and deviant. The frequency contrasts were chosen to be as close as possible to values that have been used in other studies to allow direct comparisons of the data [4], [10], [11], i.e., *Δf* = 0.37, 0.10 and 0.04; where *Δf* = (f_2_−f_1_)/(f_2_*f_1_)^1/2^ is the normalized frequency difference (Malmierca et al., 2009;Ulanovsky et al., 2003). These values correspond to frequency ratios of 0.526, 0.141 and 0.057 octaves, respectively. We quantified SSA as described previously [4], [10], [11]. The frequency-specific SSA index, SI(f_i_) (i = 1 or 2), was calculated as SI(f_i_) = [d(f_i_)−s(f_i_)]/[d(f_i_)+s(f_i_)] where d(f_i_) and s(f_i_) were responses (in spike counts/stimulus) to frequency f_i_ when it was deviant or standard, respectively. The amount of SSA for both frequencies at each condition (Common SSA index, CSI) was calculated as CSI = [d(f_1_)+d(f_2_)−s(f_1_)−s(f_2_)]/[d(f_1_)+d(f_2_)+s(f_1_)+s(f_2_)].

These indices reflect the extent to which the response to a tone, when standard, was smaller than the response to the same tone, when deviant. The indices range between −1 to +1, being positive if the response to a tone, when deviant, was greater than the response to the same tone, when standard. To thoroughly quantify the conditions that elicited SSA in a given neuron, the indices were calculated for the different combination of conditions tested (probability ratios, frequency contrasts, and repetition rates). For each neuron, this resulted in a set of SI(f_i_) and CSI values for all of the conditions that were tested.

To analyse SSA across MGB subdivisions, a fixed-effect 3-way ANOVA was performed (factors: subdivisions x Δ*f* x SOA), followed by *Post hoc* comparisons (Tukey's HSD, *p<*0.05). To analyse the effect of neuron on SSA the 3-way ANOVA was augmented into a nested design (neurons within subdivisions). To analyse the effects of neuron, Δ*f* and SOA on SSA within subdivisions, 3-Way ANOVAs were performed for each subdivision separately, with neurons considered as a random factor. All analyses were done using the statistical toolbox of Matlab (MathWorks).

### Histological verification of recording sites

Each track was marked with electrolytic lesions for subsequent histological localization of the neurons recorded. At the end of most experiments (26 out of 34) the animal was given a lethal dose of sodium pentobarbital and perfused transcardially with phosphate buffered saline (0.5% NaNO_3_ in PBS) followed by fixative (a mixture of 1% paraformaldehyde and 1% glutaraldehyde in rat Ringer's solution). After fixation and dissection, the brain tissue was cryoprotected in 30% sucrose and sectioned on a freezing microtome in the transverse or sagittal planes into 40 µm-thick sections. Sections were Nissl stained with 0.1% cresyl violet to facilitate identification of cytoarchitectural boundaries [22]. Recording sites were marked on standard sections from a rat brain atlas [65] and units were assigned to one of the three main divisions (ventral, dorsal and medial) of the MGB [22]. The stained sections with the lesions were used to localize each track mediolaterally, dorsoventrally and rostrocaudally in the Paxinos atlas [66]. To determine the three main MGB subdivisions [20], [22] cytoarchitectonic criteria, i.e., cell shape and size, Nissl staining patterns and cell packing density, were used. This information was complemented and confirmed by the stereotaxic coordinates used during the experiment to localize the MGB. After assigning a section to each track/lesion, the electrophysiological coordinates from each experiment and recording unit, i.e., beginning and end of the MGB, as well as the depth of the neuron, were used as complementary references to localize each neuron within a track. Neurons localized at the border between subdivisions and those recorded in the animals that were not perfused were excluded from this analysis. Based on selected conditions for which a large number of tested neurons were localized, topographic maps of SSA were constructed using Voronoi Tessellations of the recording sites (e.g. [36]). Each polygon was coloured according to the CSI of the unit recorded at that site. The sections shown in figure 7A were photographed at high resolution with a Zeiss Axioskop 40 microscope using a Zeiss AxioCam MRc 5 digital camera (Carl Zeiss, Oberkochen, Germany) and plan semi-apochromatic objective lenses 5× (NA 0.15). The brightness and contrast of images were adjusted with Adobe Photoshop software (Adobe, San José, CA, USA).
